# Empirical Models of Transitions between Coral Reef States: Effects of Region, Protection, and Environmental Change

**DOI:** 10.1371/journal.pone.0026339

**Published:** 2011-11-02

**Authors:** Phillip K. Lowe, John F. Bruno, Elizabeth R. Selig, Matthew Spencer

**Affiliations:** 1 School of Environmental Sciences, University of Liverpool, Liverpool, United Kingdom; 2 Department of Biology, University of North Carolina at Chapel Hill, Chapel Hill, North Carolina, United States of America; 3 Science and Knowledge Division, Conservation International, Arlington, Virginia, United States of America; Swansea University, United Kingdom

## Abstract

There has been substantial recent change in coral reef communities. To date, most analyses have focussed on static patterns or changes in single variables such as coral cover. However, little is known about how community-level changes occur at large spatial scales. Here, we develop Markov models of annual changes in coral and macroalgal cover in the Caribbean and Great Barrier Reef (GBR) regions.

We analyzed reef surveys from the Caribbean and GBR (1996–2006). We defined a set of reef states distinguished by coral and macroalgal cover, and obtained Bayesian estimates of the annual probabilities of transitions between these states. The Caribbean and GBR had different transition probabilities, and therefore different rates of change in reef condition. This could be due to differences in species composition, management or the nature and extent of disturbances between these regions. We then estimated equilibrium probability distributions for reef states, and coral and macroalgal cover under constant environmental conditions. In both regions, the current distributions are close to equilibrium. In the Caribbean, coral cover is much lower and macroalgal cover is higher at equilibrium than in the GBR. We found no evidence for differences in transition probabilities between the first and second halves of our survey period, or between Caribbean reefs inside and outside marine protected areas. However, our power to detect such differences may have been low.

We also examined the effects of altering transition probabilities on the community state equilibrium, along a continuum from unfavourable (e.g., increased sea surface temperature) to favourable (e.g., improved management) conditions. Both regions showed similar qualitative responses, but different patterns of uncertainty. In the Caribbean, uncertainty was greatest about effects of favourable changes, while in the GBR, we are most uncertain about effects of unfavourable changes. Our approach could be extended to provide risk analysis for management decisions.

## Introduction

Coral reefs are complex and diverse ecosystems with high economic and ecological value [1]. There have been substantial changes in structure and functioning of coral reef communities worldwide in recent decades [2], [3], [4], [5], and perhaps over much longer time scales [6], [7]. Coral loss has been caused by a combination of factors including global warming, land use changes that lead to sediment and nutrient pollution, overfishing, predator outbreaks, storms, and disease [8], [9], [10], [11], [12]. In some locations, mass coral mortality has led to phase shifts in which reefs have become dominated by macroalgae [2], [13], [14] or other organisms [15].

Studies of these changes generally fall into three categories. First, studies of one or a few reefs can suggest hypotheses about possible mechanisms, e.g. [2], and test these mechanisms using small-scale experiments, e.g. [16]. Second, models can be used to study the possible consequences of these mechanisms, e.g. [17], [18], [19], [20]. Third, analyses of large data sets can be used to evaluate the evidence for patterns of change over large spatial scales, e.g. [21], [22], [23].

The three approaches are complementary. Nevertheless, it may be possible to increase the value of large data sets by fitting simple dynamic models to them, combining the second and third approaches and allowing us to project the long-term consequences of current patterns of change. To date, most studies of large coral reef data sets have concentrated on describing static patterns [e.g. 24] or changes in single variables such as coral cover over time, e.g. [3], [22], [23]. For example, Bruno et al. [21] used a large database of coral and macroalgal cover on many reefs between 1996 and 2006 to examine the extent of phase shifts from domination by hard corals to domination by macroalgae. Principal component analysis was used to calculate a phase shift index, measuring the position of each reef on a scale from being dominated by corals to being dominated by macroalgae. Linear regression was then used to examine changes in the phase shift index over time. However, there is scope for analyzing the dynamics of such large data sets in more detail.

Here, we used a collection of data from coral reef monitoring studies to investigate the dynamics of transitions between coral reef states in the Caribbean and Great Barrier Reef. Our analysis is based on simple Markov models of community dynamics. Although Markov models are widely used for both marine and terrestrial communities, e.g. [25], [26], [27], [28], [29], previous Markov models of coral reefs have focussed on the small-scale dynamics of species occurrence at fixed points in space, e.g. [26], [30], [31]. Instead, we define a set of reef states (Table 1) consisting of different levels of coral and macroalgal cover, and use empirical data to estimate the probabilities of transitions between these states. Similar approaches have a long history in the analysis of vegetation dynamics, e.g. [32]. One novel aspect of our approach is that we account for the uncertainty in parameter estimates using Bayesian methods. With few exceptions [33], ecological Markov models have tended to treat parameters as if they were known exactly, when the reality is that they may be based on small numbers of observations and therefore quite uncertain. Uncertainty in parameter values carries through to uncertainty in the behaviour of the model. The Bayesian approach makes it easy to quantify this uncertainty, and thus to see whether apparent differences in the behaviour of different models are ecologically meaningful.

**Table 1:** Classification of observations into reef states (A–F) by percent cover of hard corals and macroalgae.

State	% coral	%macroalgae
A	≤25	≤25
B	≤25	25–50
C	≤50	>50
D	25–50	≤25
E	25–50	25–50
F	>50	≤50

Additionally, we asked whether there were differences in transition probabilities between the Great Barrier Reef and the Caribbean, between the first and second halves of our 10-year observation period, and between Caribbean reefs inside and outside Marine Protected Areas (MPAs). We do not examine MPA effects in the Great Barrier Reef, where only two of our sampled reefs are outside MPAs. We used our models to project the equilibrium regional pattern of reef states and cover of corals and macroalgae if conditions remain as they were during the observation period. We also examined the effects of changing the probabilities of transitions between reef states on overall coral and macroalgae cover. Such changes might result from environmental change or alterations in management. Specifically, we estimated future equilibrium reef states along a continuum of scenarios, under which we modified transition probabilities to reflect realistic changes in conditions unfavourable to coral, e.g. increased sea surface temperature [34] and those more favourable to coral, e.g. increased herbivory due to management [22], [35].

## Materials and Methods

### Data

**Figure 1:**
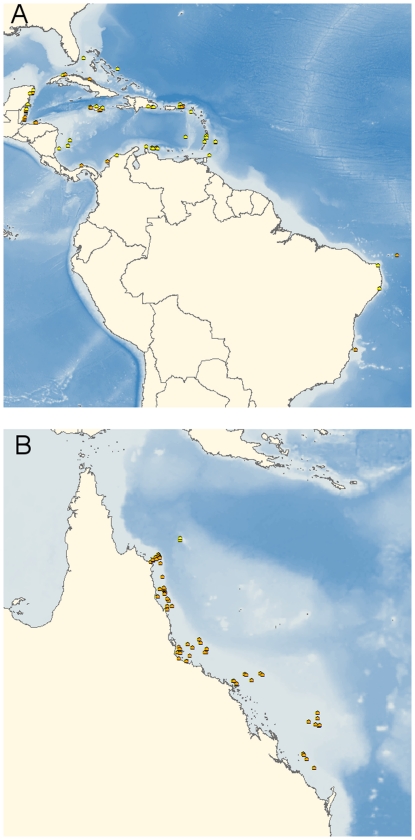
Locations of study sites in (A) the Caribbean and (B) Great Barrier Reef for which latitude and longitude data were available (not shown: 16 surveys on 8 Caribbean reefs for which there were no latitude and longitude data). Surveys within marine protected areas are shown by orange dots and surveys on unprotected reefs are in yellow.

Reef state data came from coral reef monitoring studies in the Greater Caribbean and Great Barrier Reef (GBR) regions (Figure 1) that quantified the percent of the substratum cover of living scleractinian corals and macroalgae as described in Bruno et al. [21]. All surveys were performed between 1996 and 2006. The Caribbean data set contained 100 pairs of observations on 69 reefs, while the GBR data set contained 374 pairs of observations on 55 reefs. Quantitative survey data were collected in situ using SCUBA on fore reef environments between 1 and 15 m depth (mean depth: 6.9 m). Depth explains very little of the variation in coral and macroalgal cover in these data [4](Information S1). We assume that most sites are sufficiently similar that they could in principle be in any reef state, although it would make little difference to the analysis if there were physical constraints on the states that could occur at a few sites. Surveys measured the percentage of the substratum covered by living coral and fleshy or calcareous macroalgae, primarily using some variant of the line-transect technique, in which a transect (typically a 10–30 m measuring tape or chain) was placed on the reef. The coverage of coral and macroalgae was then estimated either *in situ* by recording the number of points along each transect that overlaid corals, macroalgae, etc. or by taking images of the reef substrate at these points, which were then analyzed in the laboratory. All surveys differentiated macroalgae from other algal groups. Following [36] and others, we defined macroalgae (i.e., seaweed) as “larger (canopy heights usually >10 mm), more rigid and anatomically complex algal forms”. This functional group includes erect calcifying species (e.g., *Halimeda* spp.) but does not include microalgae and filamentous algae (i.e., turfs) or crustose algae [36]. Replicate cover measurements taken at different stations or depths at a given location were pooled into a single mean estimate for each reef in each year. Observations were made throughout the year, but we ignore seasonal variation for simplicity. We retained only those observations forming sequences of at least two observations on the same reef in successive calendar years (Caribbean: 69 reefs, median 2 observations per reef, range 2–7, covering the years 1997–2006. Great Barrier Reef: 55 reefs, median 9 observations per reef, range 2–11, covering the years 1996–2006).

Using spatial data on the boundaries of MPAs [22], [37], reef surveys that had latitude and longitude information were classified as being inside or outside of Marine Protected Areas (MPAs). For the MPA analysis, reef surveys that did not have spatial information associated with them were excluded.

### Reef state classification

Observations were classified into one of six states (labelled A to F) representing different levels of cover of coral and macroalgae (Table 1). The exact choice of category boundaries is arbitrary: we put state boundaries at 50% because it is reasonable to describe a reef with more than 50% cover of a component as dominated by that component [21], and 25% because it is halfway between zero and 50%. However, we show (Information S1) that changing these boundaries does not substantially alter our conclusions.

### Transitions between reef states

We used a simple Markov model to describe transitions between reef states. We made the following assumptions:

1. Observations on reefs are independent realizations of the same stochastic process.
2. The future state of a reef is conditionally independent of past states, given the current state.
3. The probabilities of transitions between reef states are constant over time.
4. The probabilities of transitions to each other reef state are the same for all reefs in a given state.

These assumptions are unlikely to be strictly true. For example, reefs are likely to show weak dependence through larval dispersal [38] and cover alone does not give information on variation in size structure or species composition, which may introduce historical effects that our model would not capture[30]. However, making these assumptions allowed us to use a simple modelling approach that captures some of the main features of the data.

Denote by *p_i_(t*) the proportion of reefs in state *i* at time *t*, and by *p_ij_* the probability that a reef in state *j* at time *t* will be in state *i* at time *t*+1. If we arrange the proportion of reefs in each state as a vector
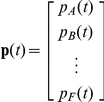
and the transition probabilities as a matrix
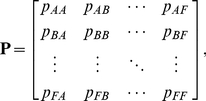
(1)then the expected proportions in each state at time *t*+1 are given by the linear equation
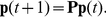
(2)Such models have been widely used for modelling successional changes in communities, especially of sessile organisms [25], [27], [28].

Dividing the continuous bivariate distribution of coral and macroalgal cover into discrete states has pros and cons. One benefit was that it allowed us to use very simple, well-understood mathematical models [25], [27], [28], and makes it easy to test hypotheses about differences between regions. Conceptually, our method is related to density-structured modelling approaches that are sometimes used in plant demography [39], [40], [41], [42], and to models in which population trajectories are classified into discrete states by direction [43]. In all these cases, discretization greatly simplifies a difficult modelling problem. On the other hand, it is likely that the discretization introduced some inaccuracy. A similar situation exists in population biology, where size structured models with discrete size classes are simple and commonly used, although models that treat size as a continuous variable are more accurate [44], [45]. We are working on related methods of modelling community dynamics that do not require discretization (K. Żychaluk et al., unpublished).

### Estimation of transition probabilities

Under our model assumptions, the numbers of transitions 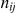 out of a given state *j* into each other state *i* have a multinomial distribution with parameters 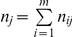 (where *m* is the total number of states, in this case six) and 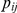, the probabilities of transition to each state *i* from state *j* (with constraints 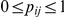 and 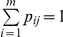). The most common approach to the analysis of Markov chain models for communities is to obtain maximum likelihood estimates of transition probabilities (described below) and calculate point estimates of derived statistics of interest [28]. However, when the transition probabilities are estimated from fairly small numbers of observations, they and any derived statistics may be subject to considerable uncertainty. Bootstrap estimates [33], [46] are unlikely to be reliable for small sample sizes. For example, if a given transition is never observed, the maximum likelihood estimate of the transition probability is zero. A bootstrap method would suggest that there is no uncertainty in this estimate, when in fact a wide range of nonzero values might be plausible.

To solve this problem, we used a Bayesian approach. Since we have no strong prior information, we used an independent uniform Dirichlet prior [47], p. 582, with parameters 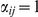 for all *i*,*j*, for the transition probabilities 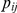 out of each state *j*. Under the resulting Dirichlet-multinomial model [47], p. 83, the posterior distribution of the transition probabilities out of state *j* is Dirichlet with parameters 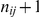 [47], p. 83. The posterior means are at 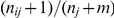 [47], p. 577, the add-one pseudocount estimates. Thus, nonzero underlying transition probabilities will not be treated as impossible even when the observed transition counts are zero.

### Estimation of initial state distribution

We needed an estimate of the initial proportion of reefs in each state to project transient dynamics. Assuming each reef is an independent realization of the same process, the state counts *n_i_(0*) at time 0 have a multinomial distribution with parameters 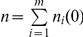 and **p**(0), a vector of state probabilities at time 0. We used a Dirichlet-multinomial model for the state probabilities, with a uniform Dirichlet prior, independent of the transition probability priors. For the Caribbean, we based our estimates on the state counts for 2006 (22 reefs). For the GBR, there were only 12 observations for 2006, so we based our estimates on the state counts for 2005 (29 reefs). We used these years, rather than the years at the start of the data set, because as the most recent data, they provide the best estimate of the current state of the reefs in each region, and are therefore appropriate for answering questions about future dynamics.

### Testing for differences in transition probabilities

Likelihood ratio tests [26], [48] can be used to test hypotheses about differences in transition probabilities between regions, time periods, or reef management categories. The maximum likelihood estimates of the transition probabilities are given by
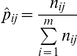

[48]. Similarly, for subsets 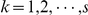 of the data, let *n_ij_(k)* denote the number of observations of a transition from state *j* to state *i* in subset *k*, and let
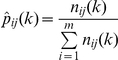
denote the maximum likelihood estimates of the transition probabilities in subset *k*. Under the null hypothesis that the transition probabilities are the same in each subset, the likelihood ratio statistic
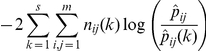
is asymptotically distributed as 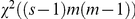 [48]. We tested three kinds of null hypothesis:

1. That the transition probabilities did not differ between the Caribbean and GBR.
2. That within each region, the transition probabilities did not differ between the first (1996–2000) and second (2001–2006) five-year periods of the decade of observations. This is one of the simplest ways of checking the assumption that transition probabilities are constant over time. Other ways of dividing the time period are possible, but not explored here. For example, we could divide the time period into a larger number of subsets, but this would result in having small numbers of observations in each subset. It would also be interesting to divide the observations into ecologically relevant subsets, based on the occurrence of region-wide disturbance events such as strong El Niño years. Again, we have not done this because it is likely that most such subsets would be small. A possible alternative is the regression approach as described in Environmental Change Scenarios below, with year and/or climatic variables as explanatory variables.
3. That within the Caribbean alone, the transition probabilities did not differ between reefs inside and outside MPAs. This allows us to investigate the effects of reef management on community dynamics. The corresponding test was not worthwhile for the GBR, where only two sampled reefs were outside MPAs.

These likelihood ratio tests do not fall within the Bayesian framework described above. However, likelihood ratio tests for problems of this kind are well-known and easy to interpret. The Bayes factors that would be used in a corresponding Bayesian analysis are somewhat harder to implement, and would require some care in the choice of an appropriate prior distribution [49, pp. 159–161]. In any case, the results of all our tests were clear-cut.

### General algorithm for derived statistics

We used the following algorithm to estimate the distribution of the derived statistics described below, for which we do not know the forms of the posterior distributions,:

1. For each of many replicates (we used 10000 in all cases):
  - Take a sample from the posterior Dirichlet distribution for each column of the transition probability matrix [47], p. 582.
  - Take a sample from the posterior Dirichlet distribution for the initial state probabilities [47], p. 582.
  - Calculate and store the value of any of the derived statistics described below.
2. The result is a sample from the posterior distribution of the derived statistic. In particular, the sample mean and symmetrical quantiles of the distribution of the derived statistic provide estimates of the posterior mean and equal-tailed credible intervals for such statistics [47], pp. 37–39. We report 50% and 95% equal-tailed credible intervals for all such statistics.

### Stationary state probabilities

As time goes to infinity, most Markov models converge to a stationary distribution of state probabilities, from any initial distribution. In our case, our Bayesian estimation method ensures that all estimated transition probabilities are positive, and so this stationary distribution is unique [50], section 4.5. This stationary distribution (which we denote by 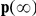) is the right eigenvector (normalized to sum to 1) of the transition probability matrix **P** corresponding to the eigenvalue with the largest magnitude [28]. The stationary distribution gives a projection of the long-term equilibrium of the system, if conditions were to remain constant. It is important to remember that this is not a forecast of what will happen, because we do not expect conditions to remain constant. Nevertheless, as in the analogous case of matrix population models, the stationary distribution is a powerful way to study the current dynamics of the system [50], pp. 29–31.

### Percentage cover of coral and macroalgae

Iterating Equation 2 allows us to calculate the projected proportion of each state at any time. However, we would also like projections of the cover of coral and macroalgae at any time. We obtain these as follows. Denote by *q_c_(t)* the projected coral cover at time *t*. Then
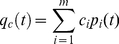
(3)where *c_i_* is the mean coral cover for reefs in state *i*. Similarly, the projected macroalgal cover *q_a_(t)* at any time *t* is
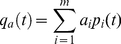
(4)where *a_i_* is the mean macroalgal cover for reefs in state *i*. We estimated *c_i_* and *a_i_* by averaging over all observations in each state *i* (we then treated these estimates as if they were known, because incorporating the uncertainty in them would require a much more complex analysis). We also obtained estimates of the stationary percentage cover of coral and macroalgae by applying Equations 3 and 4 to the stationary state probabilities 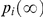, calculated as above.

### Damping ratio

The damping ratio (the ratio of the largest eigenvalue to the magnitude of the second largest eigenvalue) is a measure of the rate of approach to the stationary distribution [50], pp. 95–97. The larger the damping ratio, the more rapidly the system approaches equilibrium.

### Environmental change scenarios

To study how the system might respond to changes in environmental conditions, we need to define patterns of change in transition probabilities. Any such changes must maintain the constraints that each transition probability is between zero and one, and each column of the transition probability matrix sums to 1. Within these constraints, there are many possible patterns of change [50], p. 253. A natural choice is to consider the way we would model the response of each column of the transition probability matrix to some explanatory variable *x*. Since the counts of transitions out of each state have a multinomial distribution, the simplest statistical approach would be a baseline-category logit model [51], section 7.1:
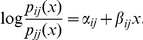
Here, we view each transition probability as a function 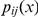 of the explanatory variable. We assume a linear model for the log of the ratio of a given transition probability to a reference probability, in this case the probability 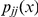 of remaining in the source state. The coefficient 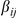 gives the slope of the response (this coefficient is zero if *i* = *j*). We represent the current conditions by 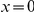 and set the intercept to 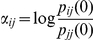 (so that the modelled transition probabilities at 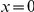 match the current estimates).

In reality, we did not have measurements of a relevant explanatory variable, so we could not estimate the coefficients 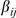. Instead, we set the signs of the coefficients (Table 2) to match what we would expect to happen as conditions change along a continuum from conditions unfavourable to coral (e.g. increased sea surface temperature) to those favourable to coral (e.g. increased herbivory). We set the magnitudes of all nonzero coefficients to be 1, as we had no information on them, and examined an arbitrary range of explanatory variable values from −2 to +2.

**Table 2:** Coefficients 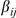 for responses of transition probabilities from state *j* to state *i* to an explanatory variable *x*, where low values of *x* represent conditions unfavourable to coral and high values represent values favourable to coral.

Change in coral cover	Change in algal cover		
	Negative^2^	Zero	Positive
Negative^1^	0	−1	−1
Zero	1	0	−1
Positive	1	1	0

1 The change in coral cover is negative if the source state *j* has higher coral cover than the destination state *i*, and vice versa.

2 The change in algal cover is negative if the source state *j* has higher algal cover than the destination state *i*, and vice versa.

### Implementation

We implemented the methods described here in Matlab R2009b (The Mathworks Inc., Natick, MA). The code is available under the GNU Public License from http://www.liv.ac.uk/~matts/coralMarkov.html.

## Results

### Distribution of reef states

**Figure 2:**
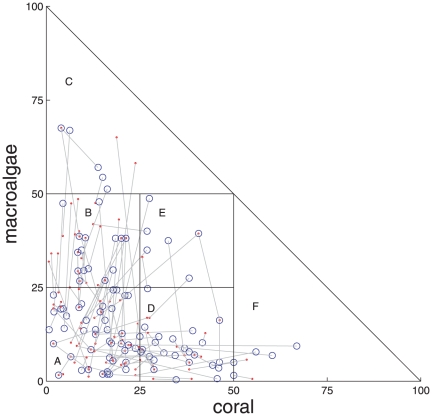
State dynamics in the Caribbean. Blue circles (year *t*)and red dots (year *t*+1), connected by grey lines, are percentage cover of coral and macroalgal cover on the same reef in two consecutive years. Black lines delimit states, as defined in Table 1. There are 100 pairs of observations in consecutive years between 1997 and 2006, from 69 reefs.

**Figure 3:**
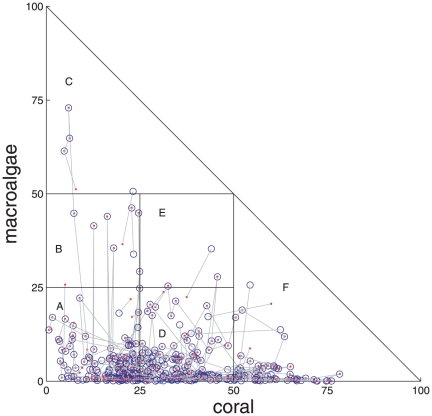
State dynamics in the Great Barrier Reef. Blue circles (year *t*) and red dots (year *t*+1), connected by grey lines, are percentage cover of coral and macroalgal cover on the same reef in two consecutive years. Black lines delimit states, as defined in Table 1. There are 374 pairs of observations in consecutive years between 1996 and 2006, from 55 reefs.

In the Caribbean (Figure 2), most observations fell in states A, B, or D (Table 1), with low to moderate cover of coral and/or macroalgae. There were frequent transitions between these states, mostly as a result of changes in either coral or macroalgal cover, but not both at the same time (Figure 2: most lines are parallel to one axis or the other). In the Great Barrier Reef (Figure 3), most observations were concentrated in states A, D, and F (Table 1), with low macroalgal cover and low to high coral cover. There was less change in reef composition from year to year in the Great Barrier Reef (Figure 3) than the Caribbean (Figure 2).

### Differences in transition probabilities between regions

**Figure 4:**
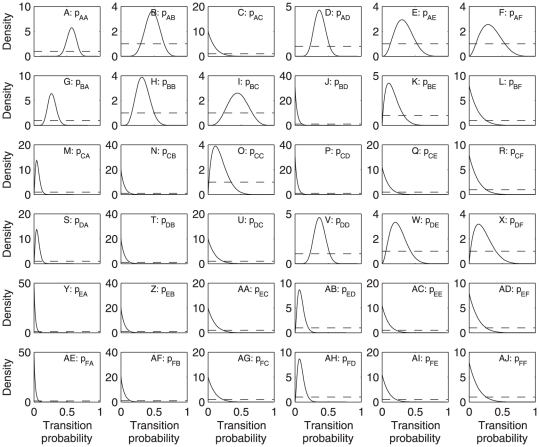
Posterior (solid lines) and prior (dashed lines) distributions of transition probabilities in the Caribbean. The panel in row *i*, column *j* is for transitions from state *j* to state *i*, as in Equation 1. See Table 1 for state definitions.

**Figure 5:**
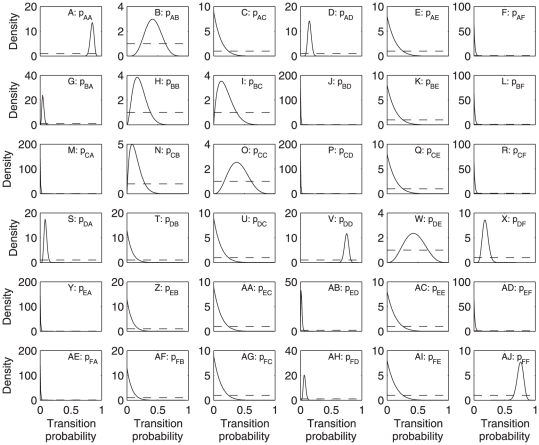
Posterior (solid lines) and prior (dashed lines) distributions of transition probabilities in the Great Barrier Reef. The panel in row *i*, column *j* is for transitions from state *j* to state *i*, as in Equation 1. See Table 1 for state definitions.

There was strong evidence for differences in transition probabilities between regions (likelihood ratio statistic 67.52, 30 df, *p* = 0.0001). Thus, we analyzed the data for the Caribbean (Table 3) and Great Barrier Reef (Table 4) separately. The transition probabilities about which we are most certain are those for which the posterior distributions have relatively sharp peaks, corresponding to large numbers of observations of the source state. In particular, we have fairly large numbers of transitions out of states A and D in both regions (Tables 3 and 4). In the Caribbean, the probability of persisting in state A, which has up to 25% cover of both corals and macroalgae, (Figure 4A) was lower than in the Great Barrier Reef (Figure 5A), while the probability of transition from state A to state B (which has up to 25% coral cover and 25–50% macroalgal cover) was higher in the Caribbean (Figure 4G) than the Great Barrier Reef (Figure 5G). Persistence in state D (25–50% coral cover and up to 25% macroalgal cover) was lower for the Caribbean (Figure 4V) than for the Great Barrier Reef (Figure 5V).

**Table 3:** Transition counts between reef states (defined in Table 1) for the Caribbean data.

	A^1^	B	C	D	E	F
A^2^	28^3^	9	0	11	3	2
B	13	6	4	0	1	0
C	2	0	1	0	0	0
D	2	0	0	11	2	1
E	0	0	0	2	0	0
F	0	0	0	2	0	0
Total^4^	45	15	5	26	6	3

1 Columns are source states.

2 Rows are destination states.

3 Cell counts are the number of pairs of observations in consecutive years on the same reef for which the first member of the pair was in the column state and the second member of the pair was in the row state.

4 Totals are the total number of pairs of observations in consecutive years on the same reef for which the first member of the pair was in the column state.

**Table 4:** Transition counts between reef states (defined in Table 1) for the Great Barrier Reef data.

	A^1^	B	C	D	E	F
A^2^	124^3^	5	0	23	0	0
B	6	2	1	0	0	0
C	0	1	3	0	0	0
D	12	0	0	119	3	12
E	0	0	0	2	0	0
F	0	0	0	10	0	51
Total^4^	142	8	4	154	3	63

1 Columns are source states.

2 Rows are destination states.

3 Cell counts are the number of pairs of observations in consecutive years on the same reef for which the first member of the pair was in the column state and the second member of the pair was in the row state.

4 Totals are the total number of pairs of observations in consecutive years on the same reef for which the first member of the pair was in the column state.

### Differences in transition probabilities between time periods and management regime

There was little evidence for differences in transition probabilities between the periods 1996–2000 and 2001–2006 in either region. In the Caribbean, the *p*-value was 0.998 (likelihood ratio statistic 12.59, 30 df, 35 pairs of observations in 1996–2000 and 65 pairs in 2001–2006). In the GBR, the *p*-value was 0.982 (likelihood ratio statistic 16.10, 30 df, 227 pairs of observations in 1996–2000 and 147 pairs in 2001–2006). In the Caribbean, there was little evidence for differences in transition probabilities between the 51 pairs of observations from reefs inside MPAs and the 43 pairs of observations from reefs outside MPAs (likelihood ratio statistic 20.92, 30 df, *p* = 0.890). Therefore, we pooled the data for both periods and for reefs inside and out of MPAs.

### Projected state and cover distributions

**Figure 6:**
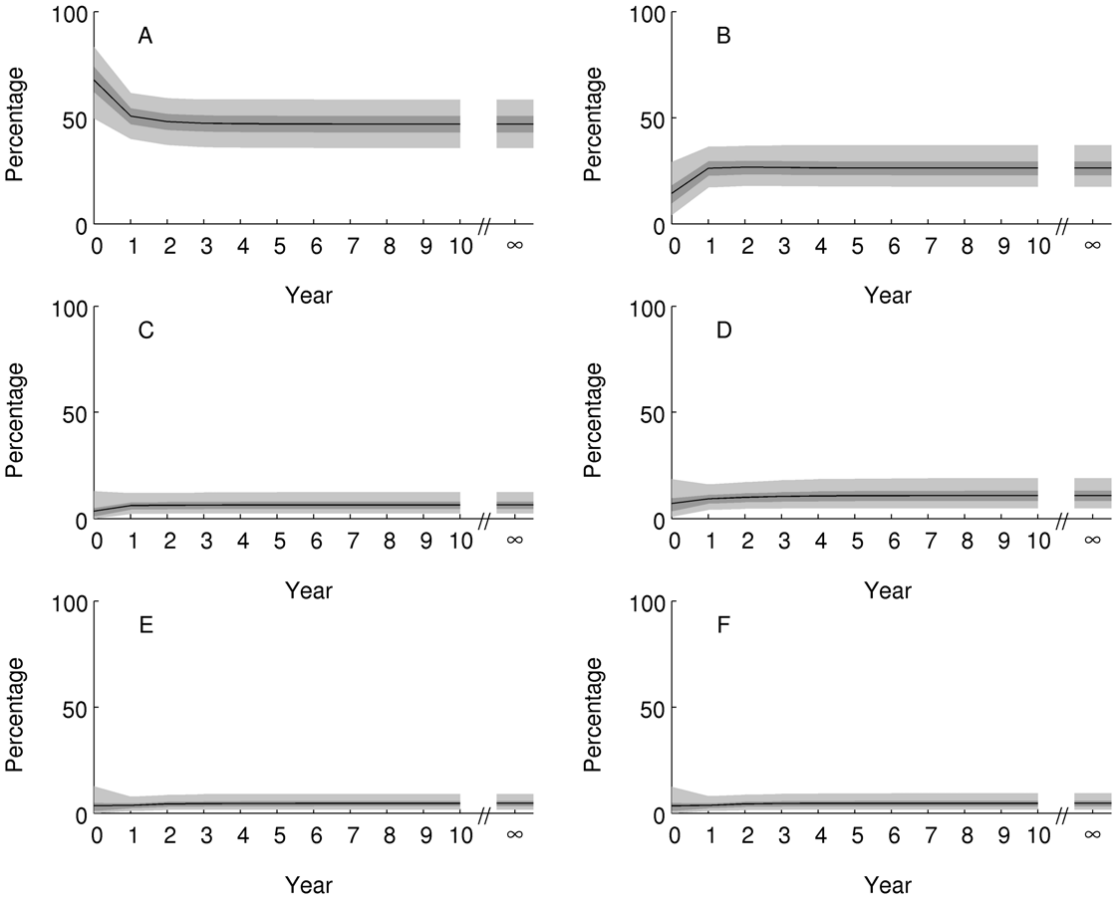
Projected percentage of Caribbean reefs in each state (panel labels match state definitions in **Table 1**) over the 10 years from 2006 (Year = 0) and at equilibrium (Year = ∞). The black line is the posterior mean, the dark shaded area is the 50% equal-tailed credible interval, and the light shaded area is the 95% equal-tailed credible interval.

**Figure 7:**
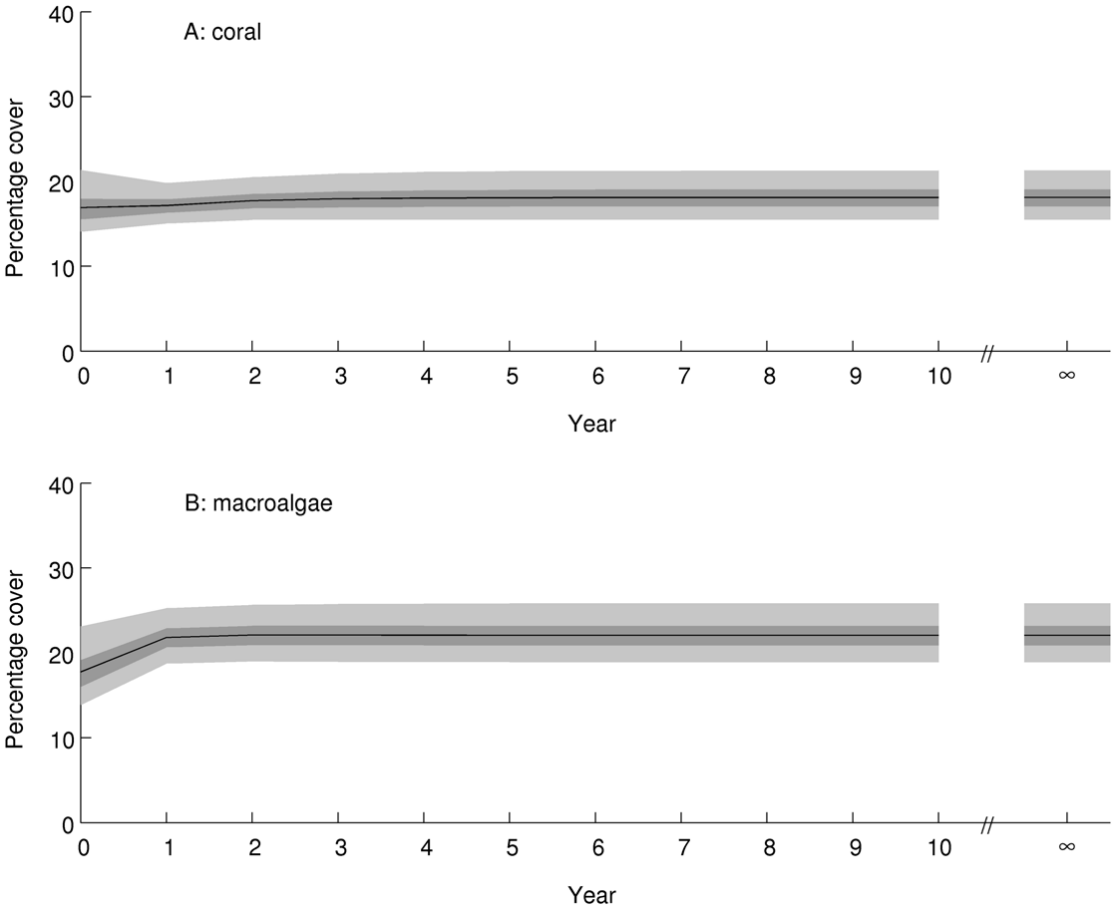
Projected percentage cover of (A) corals and (B) macroalgae in the Caribbean over the 10 years from 2006 (Year = 0) and at equilibrium (Year = ∞). The black line is the posterior mean, the dark shaded area is the 50% equal-tailed credible interval, and the light shaded area is the 95% equal-tailed credible interval.

The Caribbean system is projected to approach an equilibrium distribution within about 5 years (posterior mean damping ratio 2.55, 95% credible interval (1.78, 4.02)) if conditions remain as they were during the observation period (Figure 6). Approach to equilibrium involves a reduction in the proportion of reefs in state A (up to 25% cover of both corals and macroalgae) and an increase in the proportion of reefs in state B (up to 25% coral cover, 25–50% macroalgal cover. The other states are projected to remain relatively rare. This pattern of change is likely to be caused by the large estimated transition probability from state A to state B (Figure 4G). The overall consequence is little change in projected coral cover (Figure 7A, equilibrium posterior mean 18%, 95% credible interval (15,21)%) and an increase of a few percent in projected macroalgal cover (Figure 7B, equilibrium posterior mean 22%, 95% credible interval (19,26)%).

**Figure 8:**
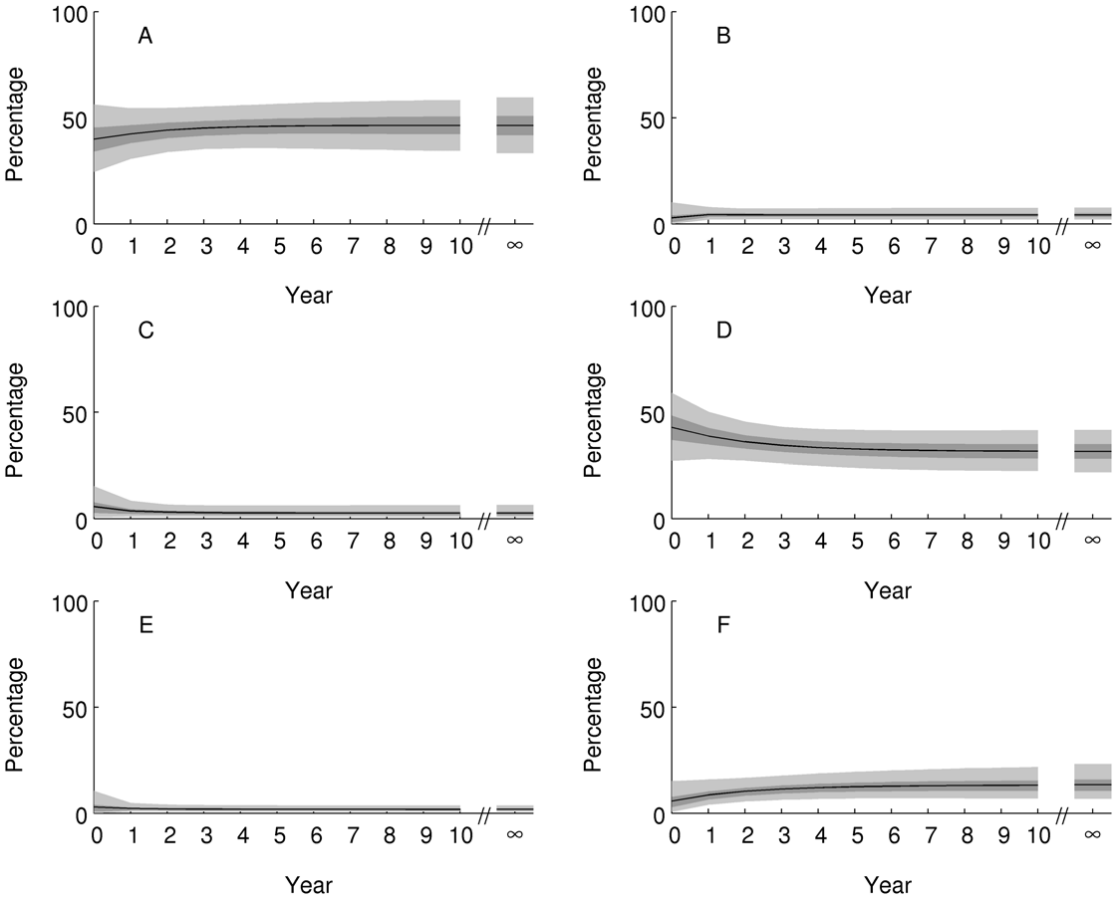
Projected percentage of reefs on the Great Barrier Reef in each state (panel labels match state definitions in **Table 1**) over the 10 years from 2005 (Year = 0) and at equilibrium (Year = ∞). The black line is the posterior mean, the dark shaded area is the 50% equal-tailed credible interval, and the light shaded area is the 95% equal-tailed credible interval.

**Figure 9:**
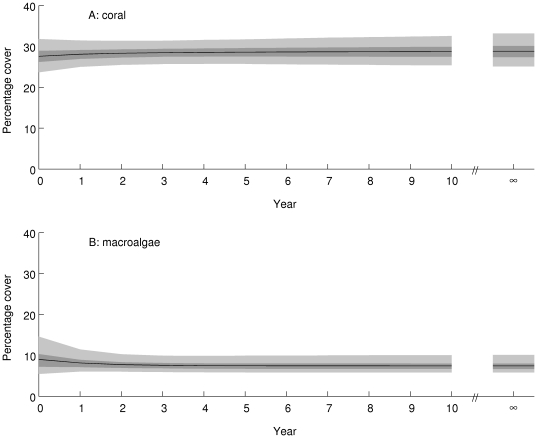
Projected percentage cover of (A) corals and (B) macroalgae in the Great Barrier Reef over the 10 years from 2005 (Year = 0) and at equilibrium (Year = ∞). The black line is the posterior mean, the dark shaded area is the 50% equal-tailed credible interval, and the light shaded area is the 95% equal-tailed credible interval.

Approach to equilibrium is projected to be somewhat slower in the Great Barrier Reef (Figure 8, posterior mean damping ratio 1.26, 95% credible interval 1.17, 1.38), with a moderate decrease in the percentage of reefs in state D (25–50% coral, up to 25% macroalgae) and a moderate increase in the percentage of reefs in state F (more than 50% coral, up to 50% algae). The overall consequence is little change in projected cover of coral (Figure 9A, equilibrium posterior mean 29%, 95% credible interval (25, 33)%) or macroalgae (Figure 9B, equilibrium posterior mean 7%, 95% credible interval (6,10)%). Compared to the Caribbean, the Great Barrier Reef is projected to maintain higher coral cover and much lower macroalgal cover, if conditions remain as they were during the observation period.

Moderate changes in the definitions of reef states do not substantially alter our projections about coral and macroalgal cover (Information S1), although they obviously alter the projected proportion of reefs in each state.

### Environmental change scenarios

**Figure 10:**
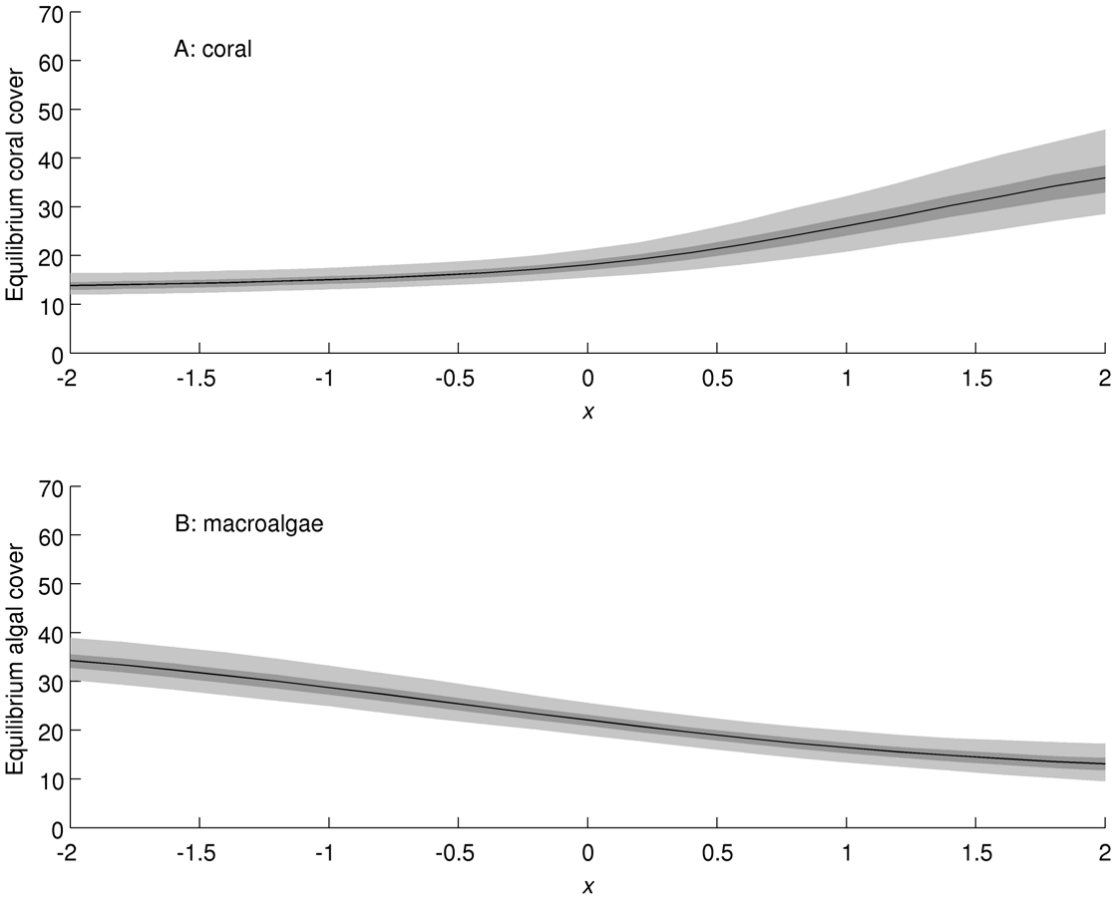
Projected responses of (A) coral cover and (B) macroalgal cover in the Caribbean to environmental change scenarios (horizontal axis: *x* = 0 corresponds to current conditions, negative values to conditions less favourable for coral, and positive values to conditions more favourable for coral). The black line is the posterior mean, the dark shaded area is the 50% equal-tailed credible interval, and the light shaded area is the 95% equal-tailed credible interval.

**Figure 11:**
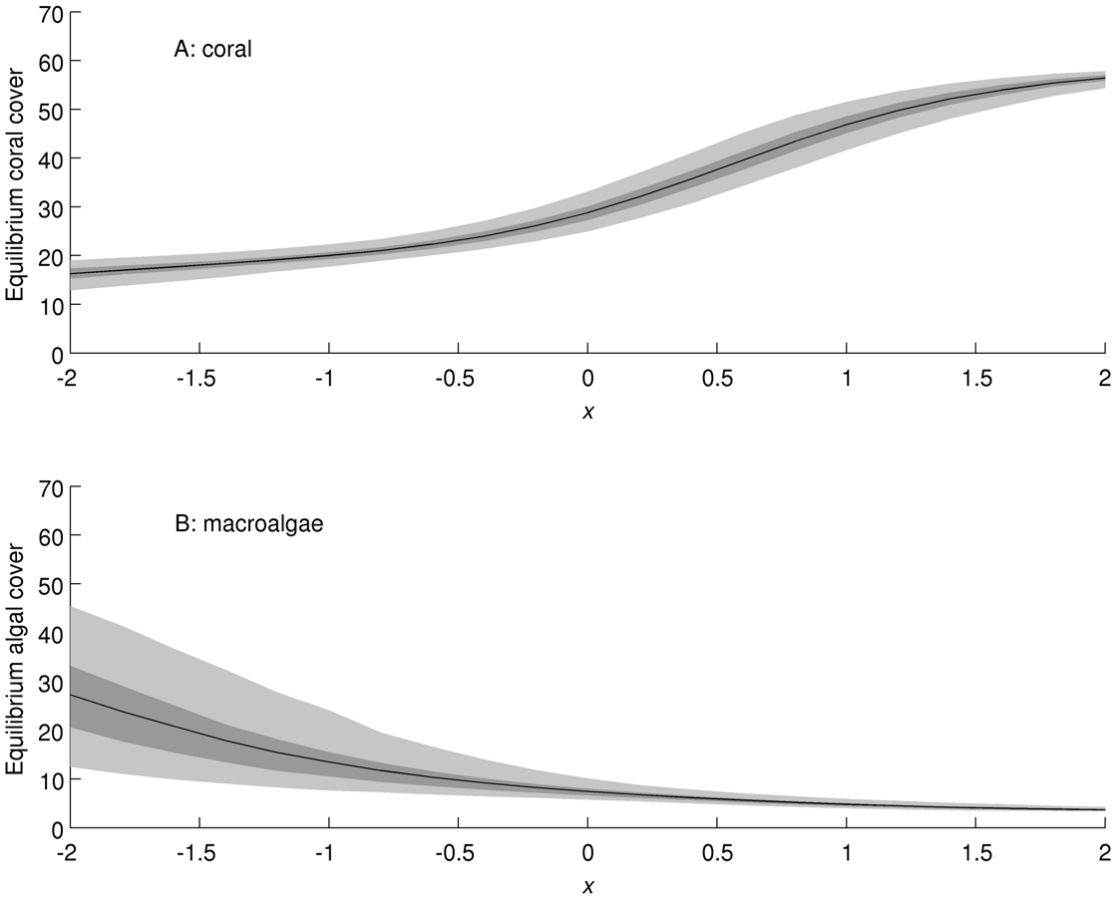
Projected responses of (A) coral cover and (B) macroalgal cover in the Great Barrier Reef to environmental change scenarios (horizontal axis: *x* = 0 corresponds to current conditions, negative values to conditions less favourable for coral, and positive values to conditions more favourable for coral). The black line is the posterior mean, the dark shaded area is the 50% equal-tailed credible interval, and the light shaded area is the 95% equal-tailed credible interval.

In the Caribbean, under conditions more favourable to coral (e.g. increased herbivory), coral cover (Figure 10A) is projected to increase and macroalgal cover (Figure 10B) to decrease, and vice versa under conditions less favourable to coral (e.g. increased sea surface temperature). The uncertainty in projected coral cover is much higher under conditions highly favourable to coral (Figure 10A, high values of *x*, an abstract measure of environmental condition). This may be because relatively few reefs were observed with high coral cover, making it difficult to project their dynamics. In the Great Barrier Reef, the responses of the posterior mean coral (Figure 11A) and macroalgal (Figure 11B) cover were qualitatively similar. However, the uncertainty in projected coral cover remains low, while the uncertainty in projected macroalgal cover becomes very high under conditions very unfavourable for corals (Figure 11B, low values of *x*). Again, this may be because we have few observations on reefs with high macroalgal cover in the Great Barrier Reef, making it difficult to project their dynamics.

## Discussion

Fitting simple models to our Caribbean and Great Barrier Reef databases of changes in coral and macroalgal cover allowed us to examine differences both in current dynamics of reefs and what these dynamics may mean for their long-term equilibria. Not surprisingly, transition probabilities were significantly different between the Caribbean and the Great Barrier Reef. This led to differences in the projected different equilibrium distributions of the proportion of reefs in each state, if conditions remain as they are now; the Caribbean has much lower coral cover and higher macroalgal cover than the Great Barrier Reef, both now and at equilibrium (Figures 7 and 9). These and other differences between these two well-studied regions are thought to be caused by a variety of factors including far more intense fishing in the Caribbean, disease outbreaks that nearly extirpated dominant coral genera and grazer species in the region [52], and possibly also the order of magnitude higher coral richness on the Great Barrier Reef [53].

Given the striking changes coral reefs in both regions underwent in the late 1970s and early 1980s (particularly in the Caribbean), transition probabilities during that period were probably very different. For example, the regional baseline macroalgal cover in the Caribbean might have been between 5 and 15% [21], and regional hard coral cover in the 1970s was around 25–50% [3], [4]. From our state definitions (Table 1), we would therefore expect that the high-algal states B, C, and E would have been rarer, and the low-algal states A, D, and F (especially D, which has 25–50% hard coral cover) would have been more common. This could have arisen from some combination of lower probabilities of transition from (A, D, F) to (B, C, E), higher probabilities of transition from (B, C, E) to (A, D, F), lower probabilities of persistence in (B, C, E), and higher probabilities of persistence in (A, D, F).

The recent regional distribution of coral and macroalgal cover is fairly close to the projected equilibrium in both regions (Figures 7 and 9). This is an important finding because so little is known about lags in the response of large scale reef dynamics to changes in the disturbance regime. The short-term impacts of recent anthropogenic disturbances, e.g., fishing and ocean warming, are well-documented, yet it remains unclear whether *past* disturbances can lead to additional *future* changes in reef state. Our estimated equilibrium distributions are projections, not forecasts, in the terminology of Caswell [50], section 2.5. We are estimating what would happen if conditions remained as they are now, not what will happen under realistic scenarios of future environmental change such as increased sea surface temperature and reduced pH [54], or under changes in the extent and enforcement of MPAs [55] . Nevertheless, such projections are useful (as they have been in population biology) because they help us to understand the current dynamics of the system. The structure of our model guarantees that the equilibrium distribution does not depend on initial conditions (Materials and Methods: stationary state probabilities). This does not imply that all reefs must end up with similar coral cover. For example, it would be possible to have an equilibrium distribution in which there were two high-probability states, of high and low coral cover, and low probabilities of transition between these states. This is the outcome we would expect from a stochastic system with alternative stable states [56].

Our results suggest that in both regions transition probabilities were not significantly different between the first and second halves of the observation period. Although it is likely that environmental conditions vary from year to year in ways that affect transition probabilities in the short term, this suggests that there were not strong or detectable trends in the underlying dynamics of the systems in the medium term. Our assumption of homogeneity when estimating transition probabilities is therefore plausible. This may seem surprising, given that the first time period includes the unusually large 1998 El Niño event, which caused substantial coral bleaching in both the Caribbean and the GBR [57]. The lack of evidence for effects on transition probabilities may be because in both regions, coral mortality was patchy rather than widespread, and recovery was generally rapid [57]. In addition, there were major bleaching events during the second time period in both regions [58], [59]. We are unlikely to have much power to detect slow long-term changes [6], given that we only have observations over a single decade. This general finding of our model is concordant with several other studies suggesting a degree of regional stasis in coral reef community composition since the mid-1980s [4], [60].

We found no evidence for an effect of protection, i.e. MPA status, on the dynamics of reefs in the Caribbean. This contrasts with other studies finding higher coral cover [61], [62] or rate of increase of coral cover in MPAs [22], [35], or lower rate of recovery from disturbance in MPAs [62], [63]. However, the sample size for this part of our study was fairly small (51 pairs of observations from reefs inside MPAs, 43 pairs from reefs not in MPAs), and it is probably more difficult to detect changes in transition probabilities than changes in overall coral cover. Thus, the power to detect effects of MPA status may have been low. Furthermore, some MPAs may be too new to affect coral cover, which can take 15–20 years to respond to MPA establishment , and not all MPAs may be managed to provide effective protection [22]. This may be partly because of the different and sometimes conflicting needs of stakeholders [64]. The level of compliance with marine reserve rules varies with socioeconomic factors, and alters the effectiveness of these reserves [65]. Effective protection may also be more difficult to achieve in marine than terrestrial reserves, because of the relative openness of marine populations, indistinct ownership boundaries in the sea, targeting of higher trophic levels for harvesting in marine than terrestrial populations, and the underlying differences in trophic structure between marine and terrestrial ecosystems [66]. These factors could contribute to the apparent absence of MPA effects in some studies [23]. In summary, MPAs would only be expected to affect transition probabilities if MPA status consistently alters key ecological processes, and this may not always be achieved.

It would be premature to conclude that MPAs do not affect the probabilities of transitions between reef states. In particular, some of the large difference in coral cover between the Great Barrier Reef and the Caribbean may be due to differences in management. For example, approximately 70% of Australian reefs, but only 10% of Caribbean reefs are inside MPAs [55], and 30% of reefs in on the Great Barrier Reef are in no-take marine reserves [67]. Although the proportions were less different in our sample (96% of GBR and 65% of Caribbean reefs in our sample are from MPAs: thus reefs in MPAs are strikingly over-represented in our samples from both regions), management may be partly responsible for regional differences in dynamics that we detected. Recent estimates suggest that the percent of protected Caribbean reefs has increased considerably [68], but reefs need to be protected for several years before benefits can be maximized [22]. The level of enforcement may also vary between the regions. In the Atlantic, 12% of MPAs were rated as effectively managed compared to 44% of MPAs in Australia [68]. It is also possible that variation in other factors such as depth, habitat type and sampling methodology between studies within a region may make it difficult to detect effects of interest in analyses of MPA effects [69], [70], [71].

Our primary analyses, e.g., projections of the equilibrium state distributions, were based on the assumption that the environmental conditions during the time the monitoring data were collected will continue into the future. However, given increases in human population growth, coastal development and fossil fuel usage, this seems very unlikely. Most marine ecologists expect the oceans of the near future to be warmer, more acidic, less productive, and even more overfished than they currently are [72], [73]. We explored how environmental changes in general might modify reef state dynamics and equilibrium state distributions by altering the state transition probabilities from the values estimated from recent monitoring data. It is impossible to know exactly how potential future changes in the environment will alter all of the transition probabilities in the matrix. Therefore, we modelled a range of possible sizes of effect, in a way that corresponds to the simplest form of environmental explanatory variable. At one extreme, the probability of transitions away from states with high macroalgal cover towards high coral cover was increased (high positive values in Figures 10 and 11). This perhaps unrealistic scenario could represent a combination of local management that effectively increases benthic grazing, thereby decreasing macroalgal cover, and coral acclimation to various local and global threats. At the other extreme are more pessimistic scenarios (negative values in Figures 10 and 11), in which we increased transitions into reef states with low coral cover and/or high macroalgal cover. This scenario could be caused by increased coral bleaching and disease as ocean temperatures continue to increase throughout the 21^st^ century.

Interestingly, the uncertainty in responses to environmental change differs between regions and between directions of change. In the Caribbean (Figure 10), uncertainty about the future state equilibrium increases as conditions become more favourable for corals, while the opposite occurs in the Great Barrier Reef (Figure 11). Intuitively, this is because change towards a situation for which we have little information increases uncertainty relative to a change towards a situation for which we have more information. For example, in the Great Barrier Reef, there were relatively few transitions observed into or out of states with more than 25% macroalgae and less than 50% coral (states B, C, and E), and so we are uncertain about the probabilities of transitions into and out of these states. If conditions change so that such states become more common, the uncertainty in these transition probabilities will have more effect on the overall uncertainty about the equilibrium distribution of the model. While the scenario analysis described here is not immediately going to provide a management tool, the underlying statistical approach [baseline–category logit models: 51, section 7.1] could in principle be used to make more realistic estimates of the effects of measured environmental variables on transition probabilities, and thus ultimately on the risk of changes in coral and macroalgal cover under environmental change scenarios. Statistical models such as the one we described here can therefore complement much more complex and mechanistic models of reef ecosystems [20], [74] as tools for ecological risk analysis.

In conclusion, the modelling approach used here allows us to detect major differences in probabilities of transitions among reef states with different levels of coral and macroalgal cover between the Caribbean and Great Barrier Reef, and to understand the consequences of those differences for the long-term behaviour of reefs in both regions. Our approach focuses on dynamics rather than current patterns, and is based on extensive data rather than hypotheses about mechanisms. Extensions of our approach that include environmental variables are likely to be useful in risk analysis for informing management decisions about coral reefs.

## Supporting Information

Information S1 **Effects of changing state definitions.**
(PDF)
