# Supplementary material for: Empirical Models of Transitions between Coral Reef States: Effects of Region, Protection, and Environmental Change
Source: PLoS One. 2011 Nov 2;6(11):e26339. doi: 10.1371/journal.pone.0026339 (PMC3206808; doi:10.1371/journal.pone.0026339)
Supplement: Information S1 — Effects of changing state definitions. (PDF) [file pone.0026339.s001.pdf]

Empirical Models of Transitions Between Coral Reef States: Effects of Regions, Marine Protected Areas, and Environmental Change Scenarios. Supporting Information S1: effects of changing state definitions

Phillip K. Lowe (primaldnb@gmail.com)

John F. Bruno (jbruno@unc.edu)

Elizabeth R. Selig (e.selig@conservation.org)

Matthew Spencer (m.spencer@liverpool.ac.uk)

As described in the main text, the continuous bivariate distribution of hard coral and macroalgal cover was divided into a set of discrete states in a way that, while not entirely arbitrary, does not necessarily correspond to natural divisions in the distribution. Thus, other choices of state definitions are reasonable. Such choices alter the proportion of reefs in each state. Here, we show that they have little effect on the projected percentage cover of corals and macroalgae.

In our original set of state definitions (Table S1, definition set 1) we put state boundaries at 50% cover, a natural choice because it is reasonable to describe a reef with more than 50% cover of some component as dominated by that component [1], and 25% (halfway from zero to the 50% boundary). Here, we examine definitions in which the 25% boundary is moved to either 15% (Table S1: definition set 2) or 35% (Table S1, definition set 3), and the 50% boundary is moved to either 40% (Table S1, definition set 4) or 60% (Table S1, definition set 5). For both the Caribbean (Figures S1 to S4) and the Great Barrier Reef (Figures S5 to S6), this makes little difference to the projected percentage cover of corals and macroalgae.

Table S1. Changes to state boundaries used to check whether the definition of these boundaries had substantial effects on projected coral and macroalgal cover. For each entry, a pair of numbers defines the percentage cover of hard corals (first number) and macroalgae (second number) for membership of the state.

	Definition set				
	1: Original	2: Figs S1, S5	3: Figs S2, S6	4: Figs S3, S7	5: Figs S4, S8
State					
A	≤25, ≤25	≤15, ≤15	≤35, ≤35	≤25, ≤25	≤25, ≤25
B	≤25, 25-50	≤15, 15-50	≤35, 35-50	≤25, 25-40	≤25, 25-60
C	≤50, >50	≤50, >50	≤50, >50	≤40, >40	≤40, >60
D	25-50, ≤25	15-50, ≤15	35-50, ≤35	25-40, ≤25	25-60, ≤25
E	25-50, 25-50	15-50, 15-50	35-50, 35-50	25-40, 25-40	25-60, 25-60
F	>50, ≤50	>50, ≤50	>50, ≤50	>40, ≤40	>60, ≤40

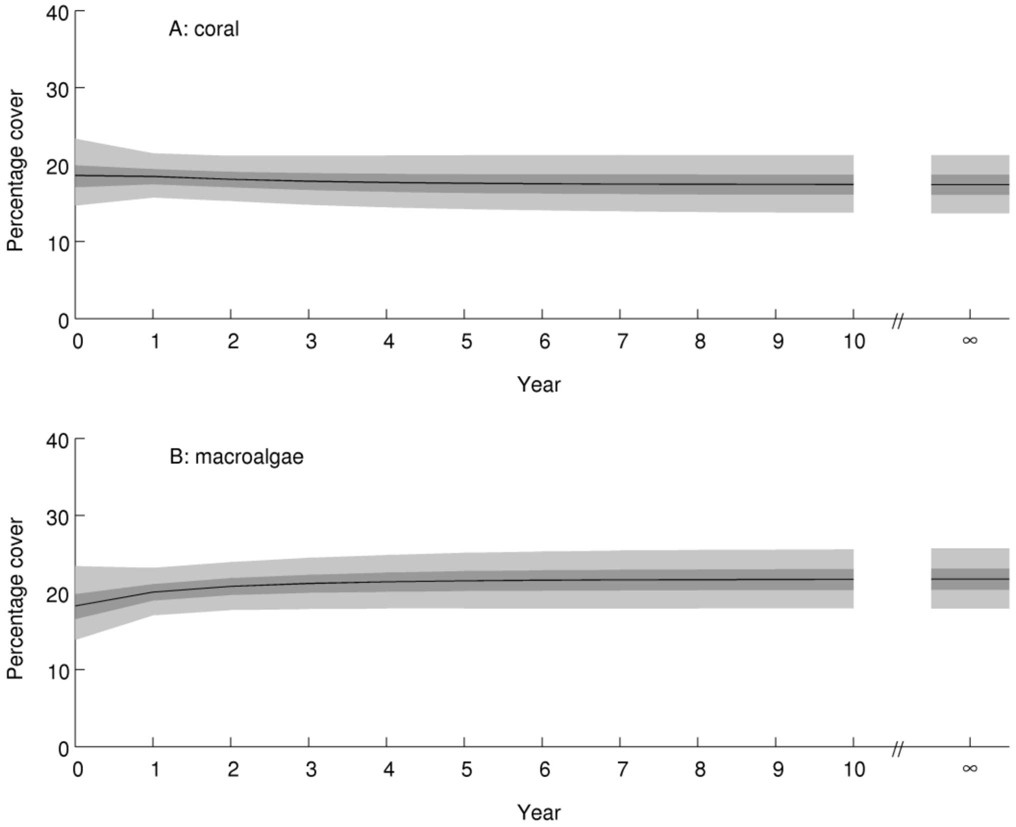

Figure S1. Projected percentage cover of (A) corals and (B) macroalgae in the Caribbean over the 10 years from 2006 (Year=0) and at equilibrium (Year=∞), with state definition set 2 (Table S1). The black line is the posterior mean, the dark shaded area is the 50% equal-tailed credible interval, and the light shaded area is the 95% equal-tailed credible interval.

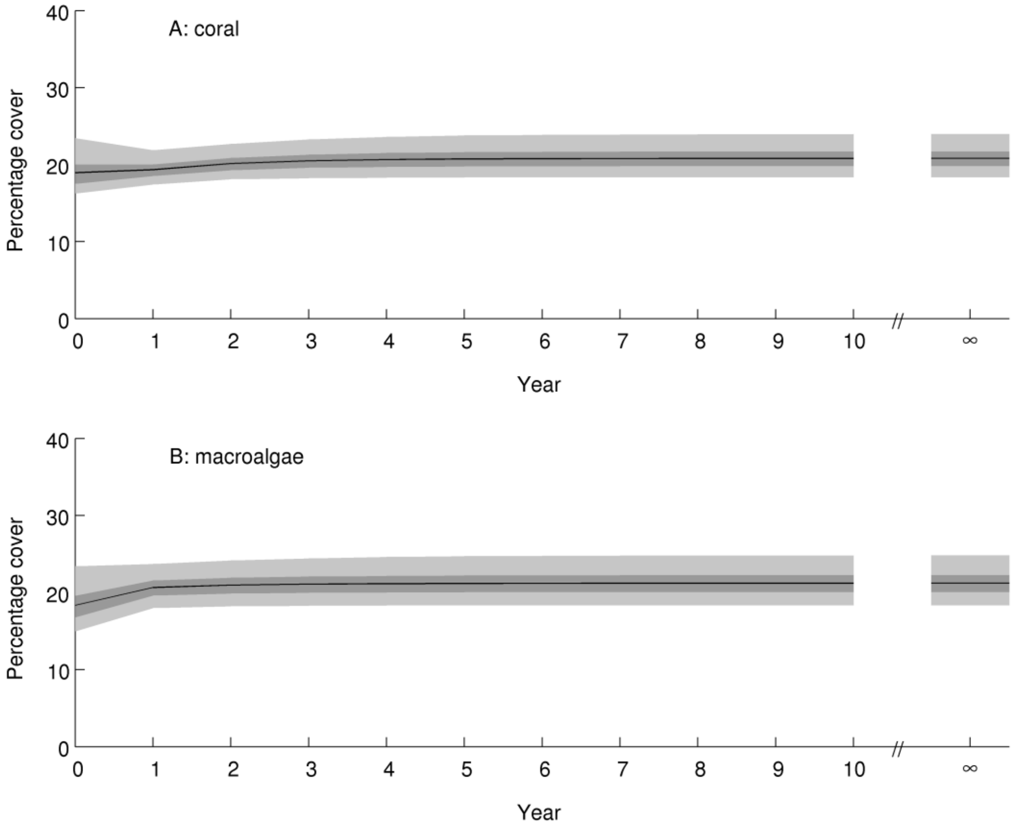

Figure S2. Projected percentage cover of (A) corals and (B) macroalgae in the Caribbean over the 10 years from 2006 (Year=0) and at equilibrium (Year=∞), with state definition set 3 (Table S1). The black line is the posterior mean, the dark shaded area is the 50% equal-tailed credible interval, and the light shaded area is the 95% equal-tailed credible interval.

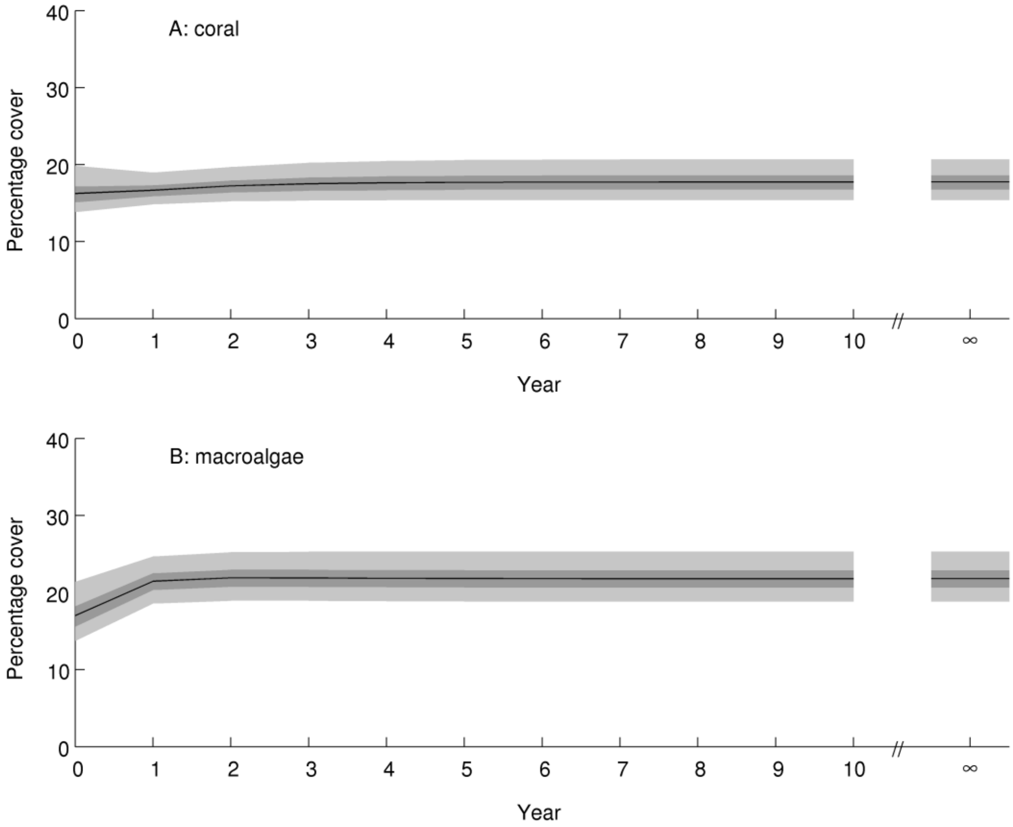

Figure S3. Projected percentage cover of (A) corals and (B) macroalgae in the Caribbean over the 10 years from 2006 (Year=0) and at equilibrium (Year=∞), with state definition set 4 (Table S1). The black line is the posterior mean, the dark shaded area is the 50% equal-tailed credible interval, and the light shaded area is the 95% equal-tailed credible interval.

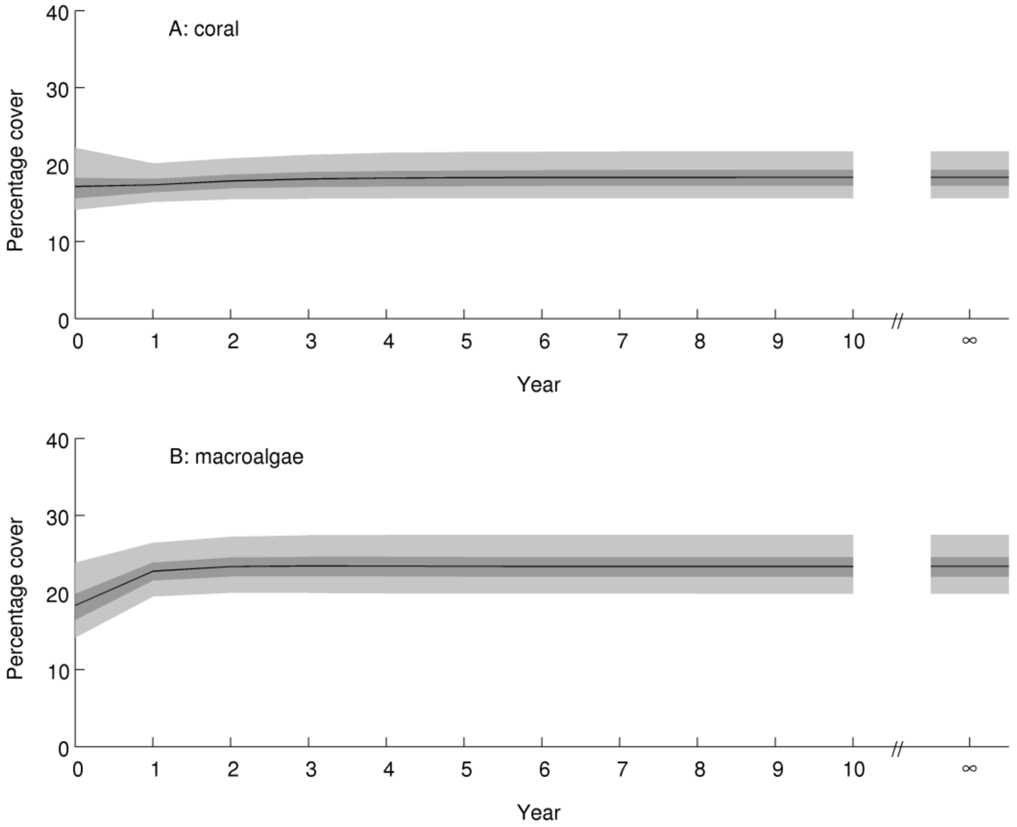

Figure S4. Projected percentage cover of (A) corals and (B) macroalgae in the Caribbean over the 10 years from 2006 (Year=0) and at equilibrium (Year=∞), with state definition set 5 (Table S1). The black line is the posterior mean, the dark shaded area is the 50% equal-tailed credible interval, and the light shaded area is the 95% equal-tailed credible interval.

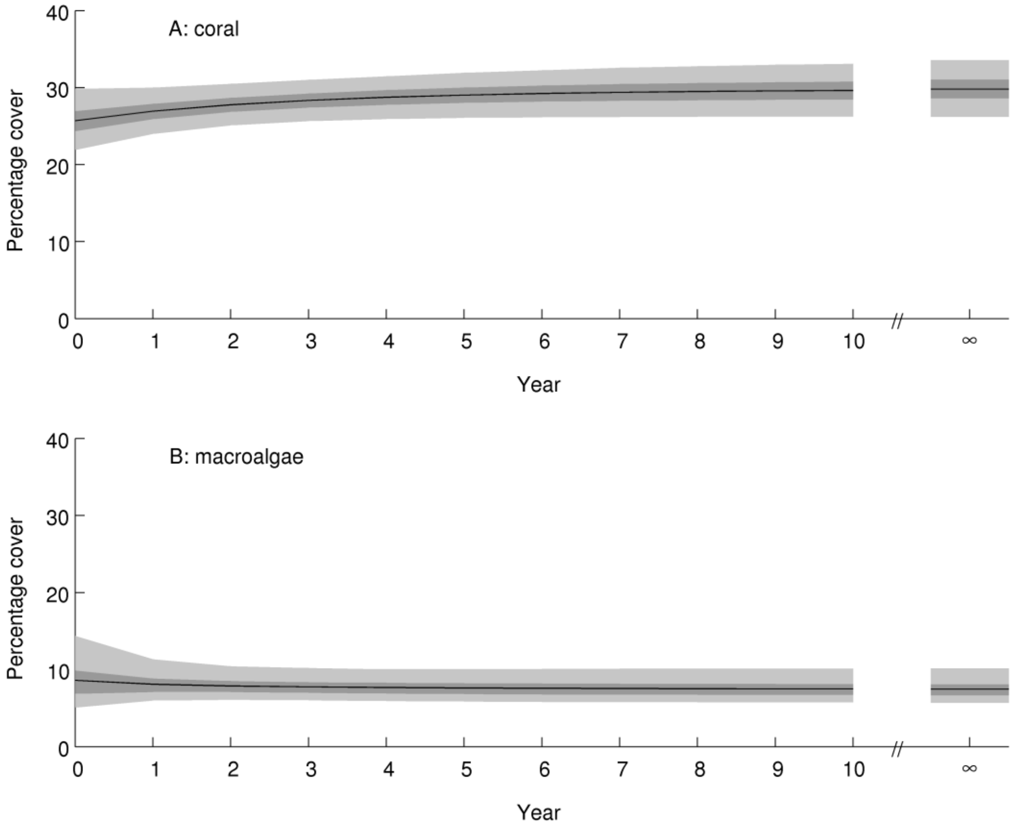

Figure S5. Projected percentage cover of (A) corals and (B) macroalgae in the Great Barrier Reef over the 10 years from 2005 (Year=0) and at equilibrium (Year=∞), with state definition set 2 (Table S1). The black line is the posterior mean, the dark shaded area is the 50% equal-tailed credible interval, and the light shaded area is the 95% equal-tailed credible interval.

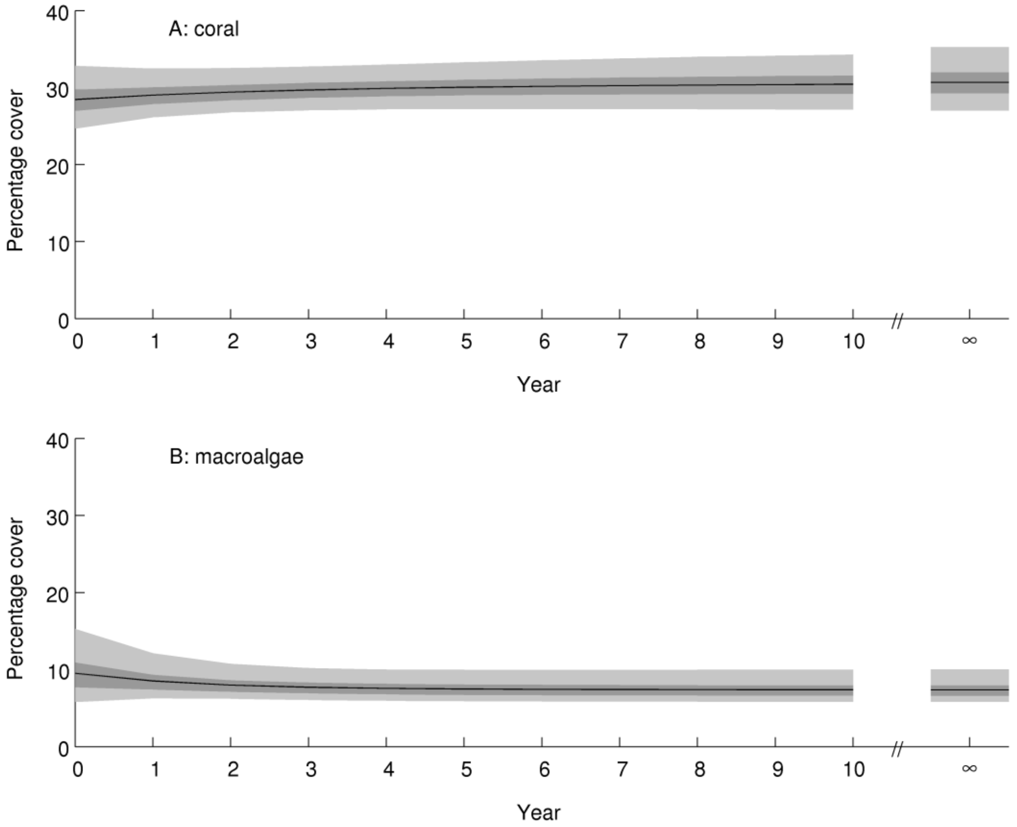

Figure S6. Projected percentage cover of (A) corals and (B) macroalgae in the Great Barrier Reef over the 10 years from 2005 (Year=0) and at equilibrium (Year=∞), with state definition set 3 (Table S1). The black line is the posterior mean, the dark shaded area is the 50% equal-tailed credible interval, and the light shaded area is the 95% equal-tailed credible interval.

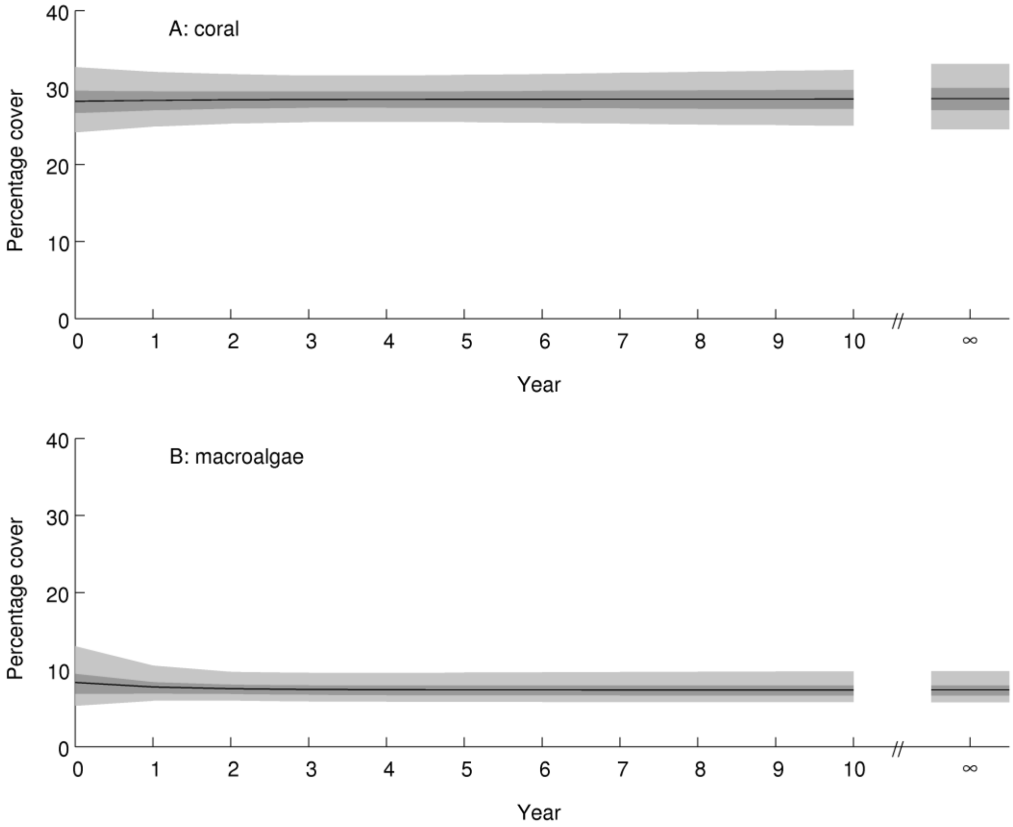

Figure S7. Projected percentage cover of (A) corals and (B) macroalgae in the Great Barrier Reef over the 10 years from 2005 (Year=0) and at equilibrium (Year=∞), with state definition set 4 (Table S1). The black line is the posterior mean, the dark shaded area is the 50% equal-tailed credible interval, and the light shaded area is the 95% equal-tailed credible interval.

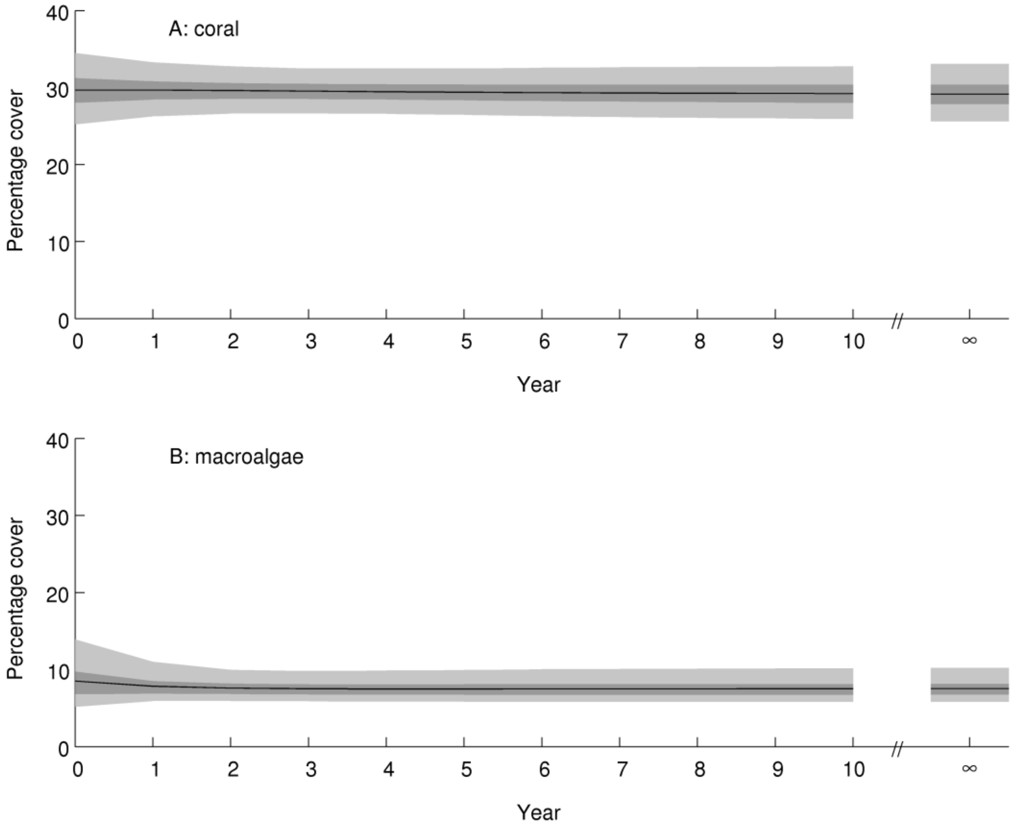

Figure S8. Projected percentage cover of (A) corals and (B) macroalgae in the Great Barrier Reef over the 10 years from 2005 (Year=0) and at equilibrium (Year=∞), with state definition set 5 (Table S1). The black line is the posterior mean, the dark shaded area is the 50% equal-tailed credible interval, and the light shaded area is the 95% equal-tailed credible interval.

References

1. Bruno JF, Sweatman H, Precht WF, Selig ER, Schutte VGW (2009) Assessing evidence of phase shifts from coral to macroalgal dominance on coral reefs. Ecology 90: 1478-1484.
